# A Matter of Contrast: Yellow Flower Colour Constrains Style Length in *Crocus* species

**DOI:** 10.1371/journal.pone.0154728

**Published:** 2016-04-28

**Authors:** Klaus Lunau, Sabine Konzmann, Jessica Bossems, Doerte Harpke

**Affiliations:** 1 Institute of Sensory Ecology, Heinrich-Heine-Universität, Düsseldorf, Germany; 2 Leibniz Institute of Plant Genetics and Crop Research (IPK), Gatersleben, Germany; Università di Pisa, ITALY

## Abstract

Most flowers display distinct colour patterns comprising two different areas. The peripheral large-area component of floral colour patterns attracts flower visitors from some distance and the central small-area component guides flower visitors towards landing sites. Whereas the peripheral colour is largely variable among species, the central colour, produced mostly by anthers and pollen or pollen mimicking floral guides, is predominantly yellow and UV-absorbing. This holds also for yellow flowers that regularly display a UV bull’s eye pattern. Here we show that yellow-flowering *Crocus* species are a noticeable exception, since yellow-flowering *Crocus* species–being entirely UV-absorbing–exhibit low colour contrast between yellow reproductive organs and yellow tepals. The elongated yellow or orange-yellow style of *Crocus* flowers is a stamen-mimicking structure promoting cross-pollination by facilitating flower visitors’ contact with the apical stigma before the flower visitors are touching the anthers. Since *Crocus* species possess either yellow, violet or white tepals, the colour contrast between the stamen-mimicking style and the tepals varies among species. In this study comprising 106 *Crocus* species, it was tested whether the style length of *Crocus* flowers is dependent on the corolla colour. The results show that members of the genus *Crocus* with yellow tepals have evolved independently up to twelve times in the genus *Crocus* and that yellow-flowering *Crocus* species possess shorter styles as compared to violet- and white-flowering ones. The manipulation of flower visitors by anther-mimicking elongated styles in *Crocu*s flowers is discussed.

## Introduction

Flowers are signalling apparatuses that attract pollinators and repel antagonists by means of their combination of visual, olfactory, gustatory and tactile/mechanical signals [1,2]. The overall flower colour is thought to be a main attractant at long distances [3], whereas floral guides, sometimes referred to as nectar guides or honey guides, guide approaching flower visitors at close range [4–9]. Most floral guides are yellow-coloured, absorb ultraviolet light, and in this way mimic the colour of pollen and anthers [10–12]. Pollen eating flower visitors possess innate search images (sensu Menzel [13]) for pollen-bearing stamens and naïve individuals respond to stamens as well as to stamen-mimicking structures [4,6,14–17].

**Fig 1:**
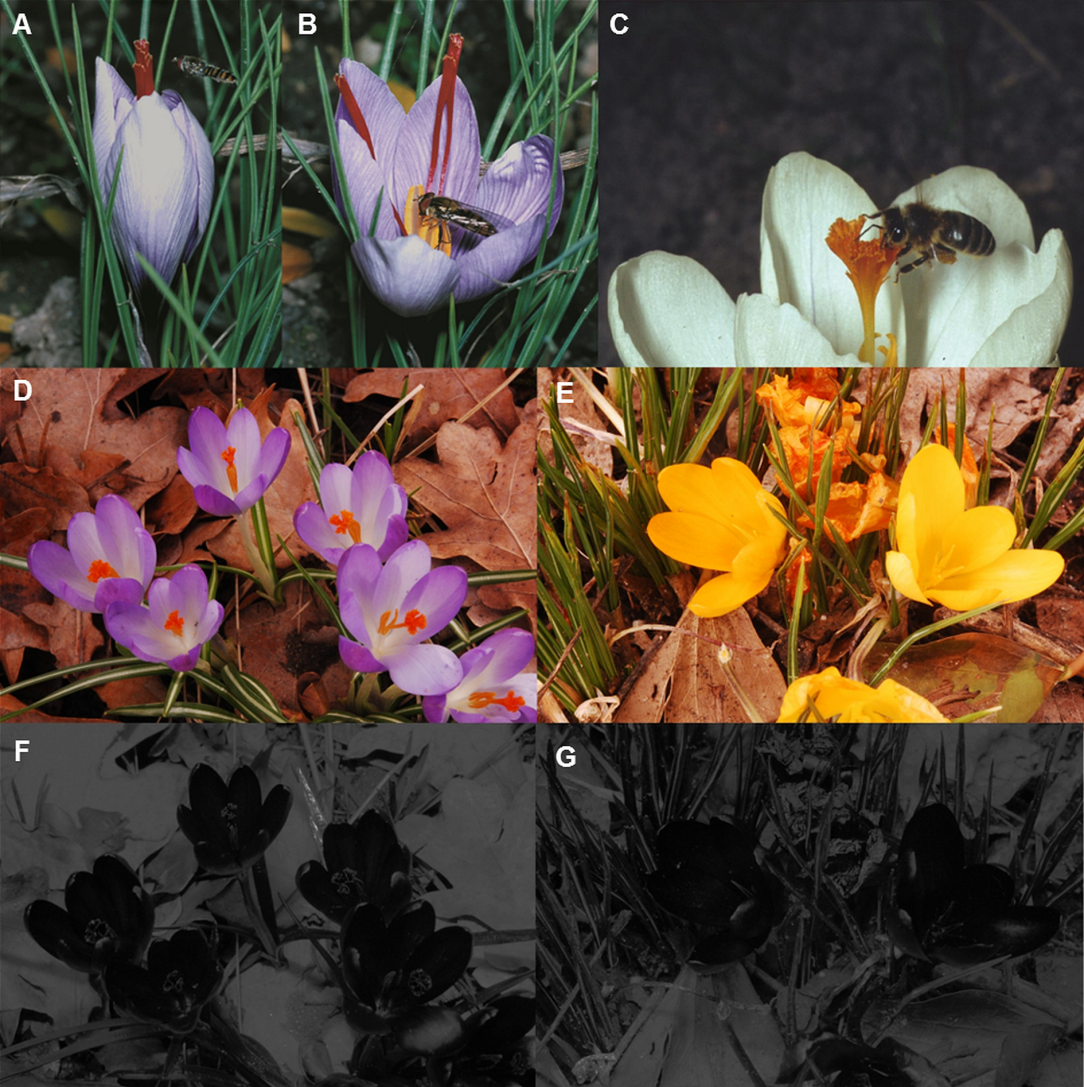
Flowering phases and UV-reflectance properties of *Crocus* species. (A) Flower of the saffron crocus *C*. *sativus* in the early flowering phase with a syrphid fly, *Episyrphus balteatus*, targeting at the stigma. (B) The same flower in the subsequent flowering phase with a syrphid fly feeding on pollen. (C) Western honeybee, *Apis mellifera*, landing on a *Crocus* flower and targeting at the stigma. (D–G) Colour photograph and UV-photograph of violet and yellow garden crocuses.

Even experienced honeybees show antennal reaction at pollen-yellow floral guides [14]. The latter are also one of the main pollinators for species of the genus *Crocus* (Iridaceae) flowering in spring or in autumn [18]. Cross-pollination seems to be necessary for the generative propagation in *Crocus* as for most of the up to now investigated species of the genus self-incompatibility has been stated [19,20]. Besides, cross-pollination may also be facilitated by spatial distance between style and stamens. The violet flowers of *C*. *etruscus* are self-incompatible, but still feature spatially separated reproductive organs [21], which indicates an additional relevance of differing lengths of style and stamens. *Crocus* flowers possess rather uniform solitary, cup-shaped, radially symmetrical, hermaphroditic flowers tapering off into a narrow tube. The overall flower colour of the six tepals varies among species and includes violet, yellow and white; most *Crocus* flowers are dominated by one tepal colour, sometimes possessing patterned tepals. Members of the genus *Crocus* typically have three mostly yellow to orange stamens offering likewise yellow or orange pollen [22]. The style of most species has three stigmatic lobes, varying in length among species, but possesses a similar colour as compared to the pollen bearing anthers. Particularly in the saffron crocus, *Crocus sativus*, the style is elongated and conspicuously red coloured due to highly concentrated carotenoid pigments [23]. In some species of the genus, each branch of the style is ramified or the tips of the stigmatic lobes are broadened and thus visually very conspicuous. Due to their colour and form, the three stigmatic lobes of some *Crocus* species are supposed to mimic the anthers, which in combination with the spatial arrangement of the stigmatic lobes direct approaching and landing bees or hoverflies towards the stigma [12]. In this case, the flower visitors touch the stigma prior to any other part of the flower, thus improving cross-pollination (Fig 1A–1C).

Previous studies have shown how conspicuous parts of the colour patterns of flowers, i.e. floral guides, contribute to direct approaching bees towards the site of floral reward [6,14] and that the within-flower colour contrast plays an important role for the detection of floral guides [24]. It is noteworthy that researchers use the term colour pattern in different ways, namely as a spatial pattern originating from bi- or multi-coloured tepals or petals [25–27], or–as in this study–as a spatial colour pattern of flowers irrespective of the organs involved such as petals or tepals, stamens and style [28,11,12].

Not all *Crocus* species display an elongated style as compared to the anthers and thus resign the possibility to direct landing flower visitors towards the stigmatic lobes. Yellow-flowering species might be less successful in directing the flower visitors towards the yellow style and accordingly use a deviant pollination strategy in which the prompt disposal of pollen from other flowers on the stigma is less important. We ask whether the within-flower colour contrast between style and corolla constrains the display of conspicuous and attractive landing sites and hypothesise that the colour contrast between style and tepals in yellow flowering species of the genus *Crocus* is less strong than in violet or white ones. Since all potential pollinators, i.e. bees and flies, are able to see ultraviolet light, our hypothesis predicts that the ultraviolet reflectance of tepals and stigma in yellow flowering *Crocus* species is small. Because many yellow bee-pollinated flowers display a UV-reflecting periphery and a UV-absorbing centre [29,12], the so-called bull’s eye pattern [30], it is claimed that *Crocus* flowers do not rely on an ultraviolet colour pattern. Indeed, UV-photographs of some *Crocus* flowers [31] and spectral reflectance data of tepals in some species indicate that the tested yellow-flowering species possess an ultraviolet absorbing perigone, which does not provide strong colour contrast against the likewise yellow and UV-absorbing stigmas and stamens [32]. Moreover, a survey of ultraviolet reflectance properties in the genus *Crocus* showed that all 39 species studied so far, including 24 violet-, 9 white-, and 6 yellow-flowering species (*C*. *ancyrensis*, *C*. *boryi*, *C*. *chrysanthus*, *C*. *flavus*, *C*. *korolkowii*, *C*. *olivieri*) have ultraviolet absorbing flowers (less than 5% UV-reflectance) and display no UV-reflectance pattern; 80% of the species were classified as having very strong or strong UV-absorption [25].

Assuming that the colour contrast between style and perigone represents a prerequisite for the attraction and guidance of bees towards the stigma, we hypothesize that elongated styles are more frequent among violet and white as compared to yellow-flowering *Crocus*.

In order to test this hypothesis we calculated the relative length of stamens and styles in relation to the length of the tepals for yellow, violet and white *Crocus* flowers using photographs, mainly from internet sources. We mapped our data onto a phylogenetic tree of the genus *Crocus* in order to exclude phylogenetic bias. In addition, we took UV-photographs and measured the spectral reflectance properties of the majority of yellow-flowering *Crocus* species in order to check the bee-subjective colour pattern provided by the flowers.

## Materials and Methods

A phylogenetic analysis of the internal transcribed spacer region (ITS: ITS1, 5.8.S, ITS2) of the nuclear ribosomal DNA was carried out with 106 species of the genus *Crocus* representing all major phylogenetic groups within the genus and 3 species of the genus *Romulea* as an outgroup. All sequences were generated in previously published studies [33–35] and subjected to phylogenetic analyses using Bayesian phylogenetic inference (BI) with MRBAYES 3.2 [36] running for 6 million generations. We analysed the resulting phylogenetic tree of the *Crocus* and *Romulea* species of which we gathered images, including 13 photographs that were taken, 95 colour photographs from 10 different internet sources and 1 photograph from a species description [37]. These images (origin of photographic material listed in S1 Table) were used to categorise the overall flower colour, the colour of style and stamens, the length of style and stamens in relation to the tepal length, and the form of the style of the 109 species considered. The relative stamen length was plotted against the relative style length for members of the genus *Crocus*. The overall flower colour was categorised as “yellow”, “white”, “violet” and “violet or white”. Species exhibiting colour polymorphism, e.g. *C*. *cvijicii* and *C*. *danfordiae*, were categorised according to their most common perigone colour. The relative length of stamens and style was categorised as “stamens longer than style”, “stamens as long as style” (equal length) and “stamens shorter than style”. Information on style-stamen length ratio was verified for all investigated species using the original descriptions or the information given in Maw [38] and Mathew [22]. The form of the style was categorised as “three-lobed” and “highly branched”. These data were mapped onto the phylogenetic tree.

UV-photographs of some *Crocus* flowers were taken by using a UV-sensitive Nikon D60 (Tokyo, Japan) camera or a Lumix GH1 camera equipped with a UV-permeable quartz glass lens (Ultra-Achromatic-Takumar 85mm; Asahi Optical Co., Tokyo, Japan), which was then combined with a filter transmitting human-visible light (UV ⁄ IR cut filter: Baader UV-IR-Cut ⁄ L-filter) or a UV-transmittance filter (Baader U-filter 2, transmission peak wavelength 350nm, half-bandwidth 60nm; Baader Planetarium, Mammendorf, Germany) in daylight.

The spectral reflectance of *Crocus* tepals was measured with the spectrophotometer JAZ Spectrometer System (Ocean Optics, Inc., Florida, USA) at an angle of 45° to the measuring spot, which was combined with a fibre-optic cable (UV-VIS 400μm; World Precision Instruments, Inc., Florida, USA). A pellet of barium sulphate powder was used as white standard and a black film can as black standard.

The proportion of species in which the style length surpasses the stamen length was tested by means of a two-sided Fisher’s Exact Test. A linear regression analysis of the phylogenetic independent contrasts [39] was conducted to test the correlation between the lengths of stamens and style.

## Results

**Fig 2:**
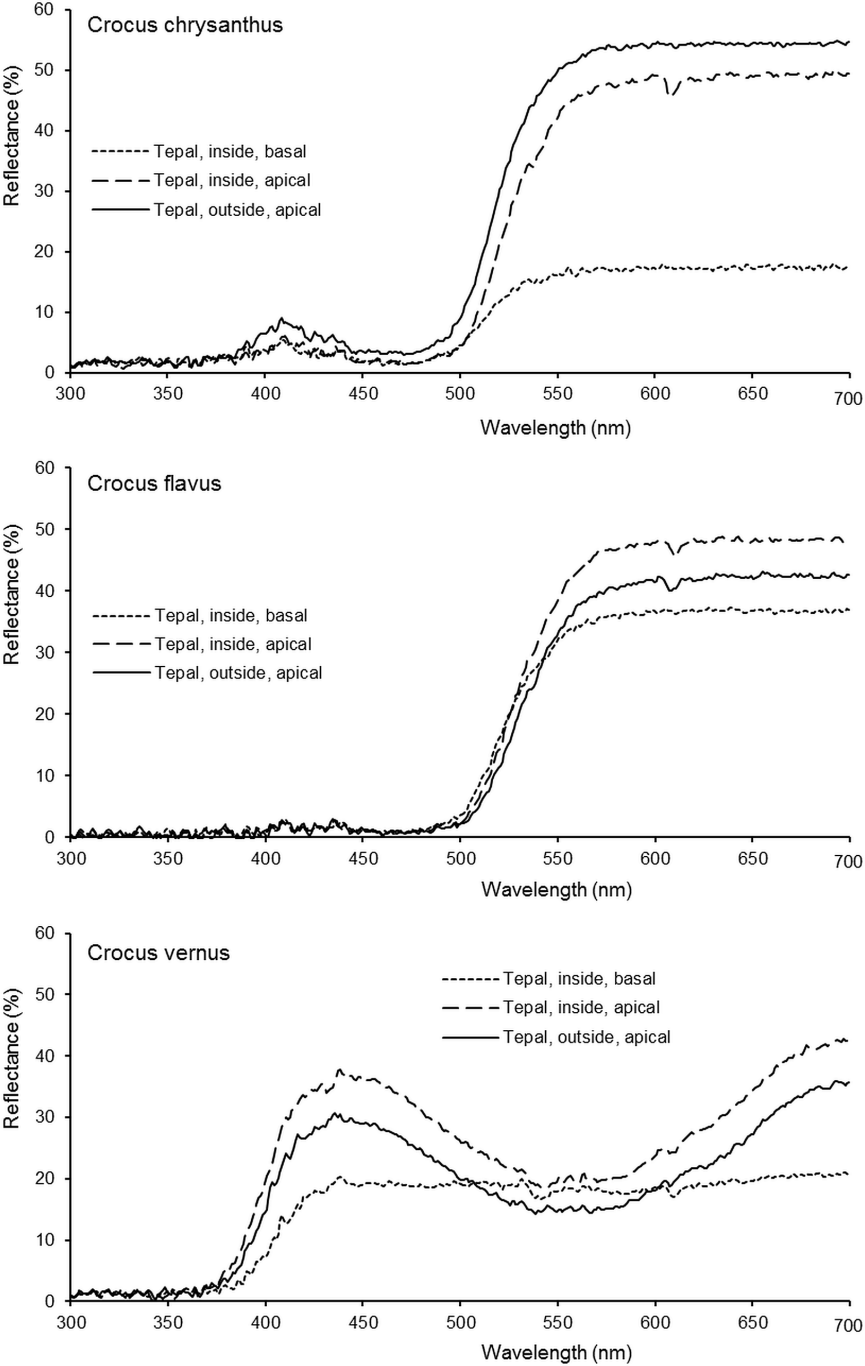
Reflectance properties of differently coloured *Crocus* flowers. Relative spectral reflectance of tepals in the yellow-flowering *C*. *chrysanthus* and *C*. *flavus* and the violet- or white-flowering *C*. *vernus*.

**Fig 3:**
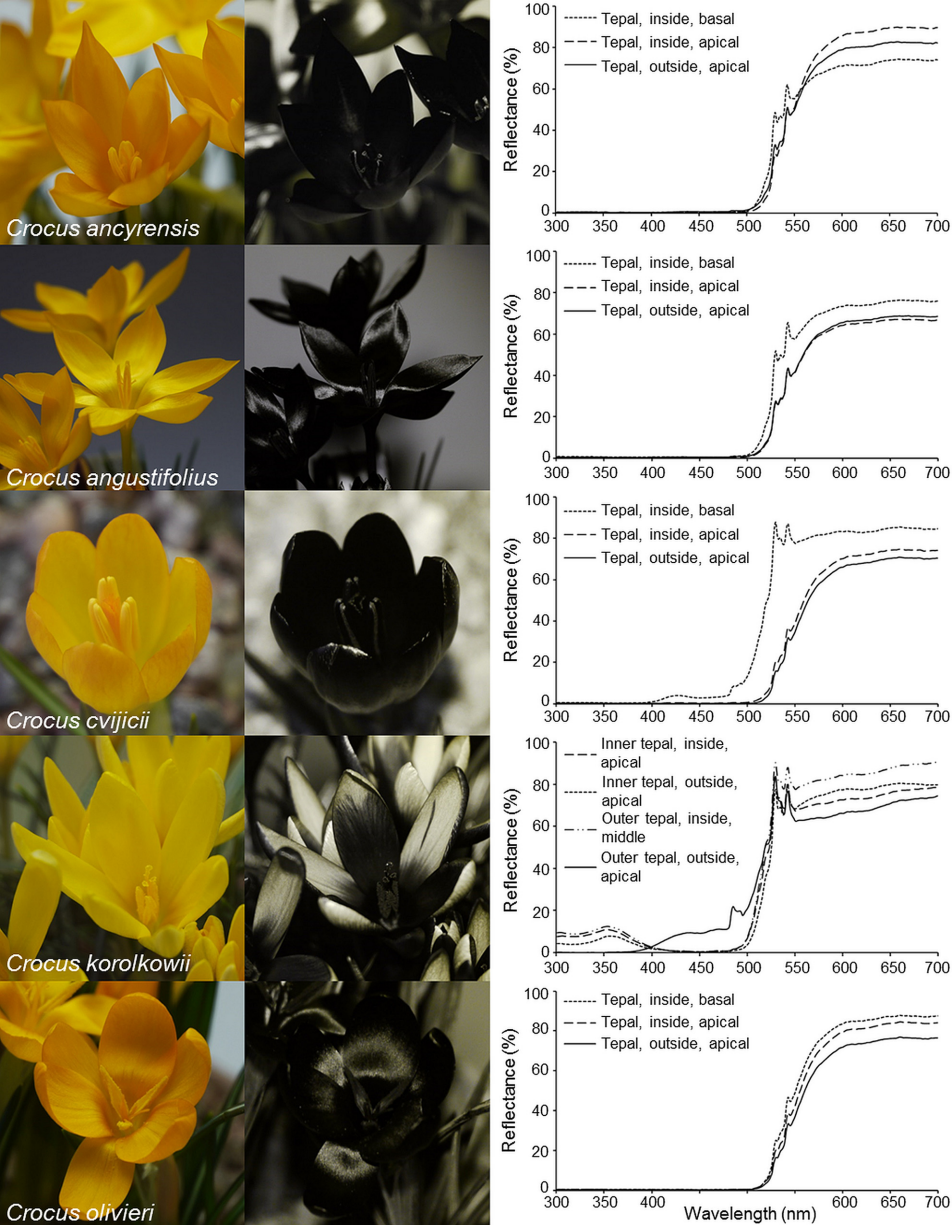
Comparison of reflectance properties of some yellow-coloured *Crocus* species. Colour photograph, UV-photograph and relative spectral reflectance of tepals are shown for five exemplary yellow-flowering *Crocus* species. Note that only in *C*. *korolkowii* parts of the tepals are distinctly UV-reflecting.

The ultraviolet reflectance properties of some violet- and most yellow-flowering *Crocus* species are rather uniform: For example, the flowers of violet and yellow garden crocuses do not display any UV-pattern and strongly absorb ultraviolet light in all floral organs (Figs 1D–1G and 2). Similarly, all floral organs of the yellow-flowering *Crocus* species *C*. *ancyrensis*, *C*. *angustifolius*, *C*. *chrysanthus*, *C*. *cvijicii*, *C*. *danfordiae*, *C*. *flavus*, *C*. *gargaricus*, *C*. *graveolens*, *C*. *olivieri*, *C*. *sieheanus*, *C*. *thirkeanus*, and *C*. *vitellinus* strongly absorb ultraviolet light, with only one exception: In *C*. *korolkowii* the three inner perigone leaves strongly absorb ultraviolet light, whereas the three outer perigone leaves reflect some ultraviolet light (Fig 3). The spectral reflectance properties of the inside and the outside of the tepals of some species confirm these findings, particularly the strong absorption in the ultraviolet range of wavelengths in the yellow-flowering species (Fig 2). The particular example of the saffron crocus shows how a long style might facilitate pollen transfer between flowers of the staminate towards flowers of the pistillate flowering phase by displaying the sole ‘floral guide’ at the beginning of the anthesis of each individual flower, when only the style, but not the stamens are visible in the just opening flower; later the stamens replace the style as an attraction signal and landing site (Fig 1A and 1B). The saffron crocus is male sterile [40]; however, in fertile species like the closest relative of saffron, *C*. *cartwrightianus*, this kind of pollen transfer may improve cross-pollination. Another photograph shows a white *Crocus* flower offering a landing site for a honeybee in the form of an elongated style (Fig 1C).

In our study, 106 *Crocus* species were included for which stamen and style length in relation to tepal length was analysed with regard to perigone colour.

**Fig 4:**
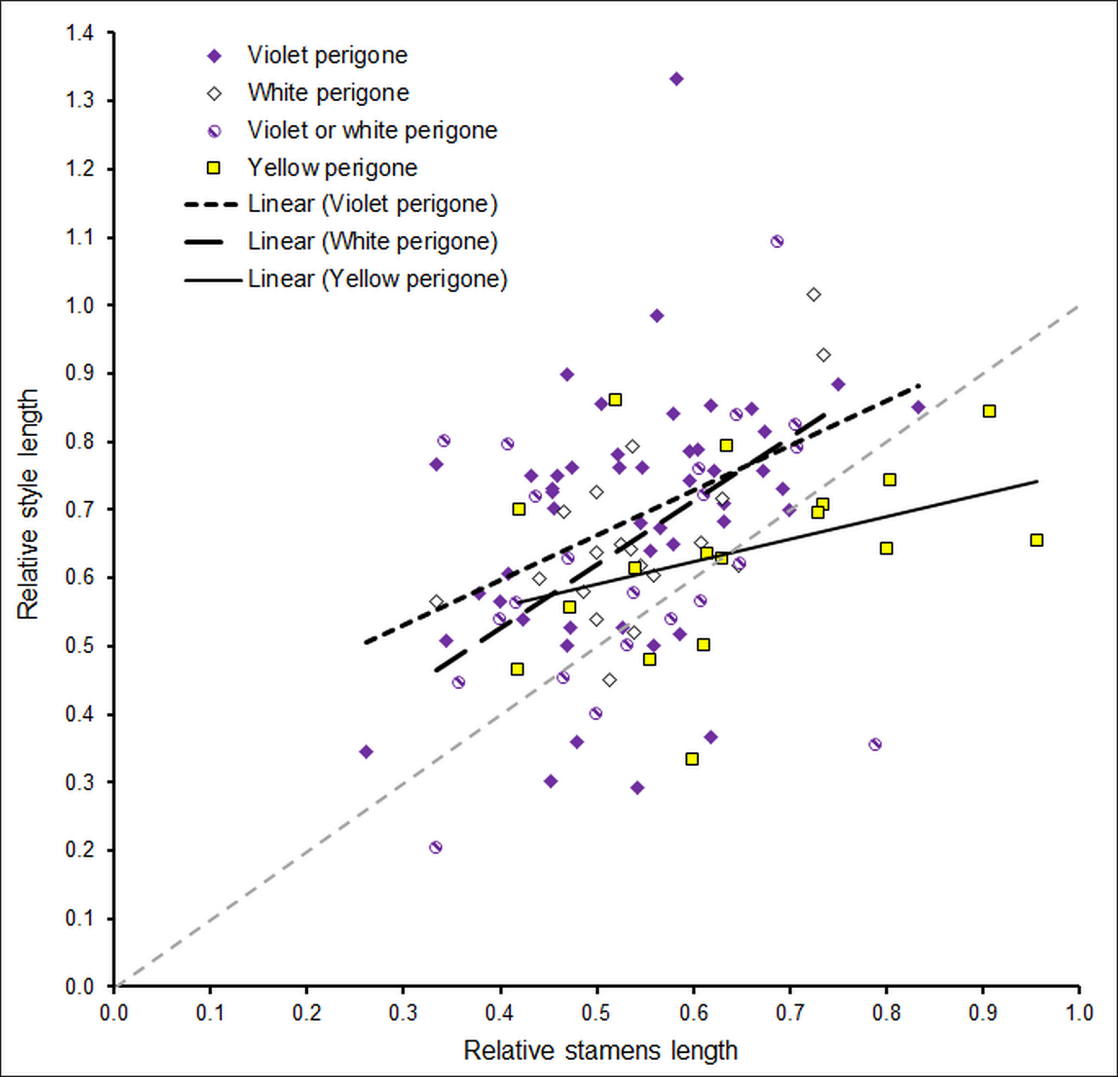
Style length dependent on stamens length in relation to perigone colour. Analysis of 106 *Crocus* species regarding perigone colour. Both style length and stamens length are relative to the length of the tepals.

In 77 out of 106 tested *Crocus* species (75.5%), the style length surpasses the stamen length. In 26 species (25.5%), the stamens are longer than the style. In three species (2.9%), the style and stamens do not differ in length (Table 1). The length of the style in relation to the length of the tepals is similar for *Crocus* species of different flower colour, 0.64±0.14 in yellow (n<17), 0.66±0.13 in white (n = 19), 0.62±0.19 in white/violet (n = 22), and 0.69±0.19 in violet species (n = 48). The style length surpasses the tepal length in only three species, none of which is yellow-flowering (Fig 4). In 70 of the 89 non-yellow-flowering *Crocus* species (78.7%) the style length surpasses the stamen length, whereas in only 7 of the 17 yellow-flowering *Crocus* species (41.2%) the style is longer than the stamens (Table 1; p = 0.0030, two-sided Fisher’s Exact Test).

**Table 1:** Longest reproductive organ in *Crocus* species. Numbers of differently coloured *Crocus* species in which the style length surpasses the stamens’ length, vice versa, or both organs have the same length.

Perigone colour	Number of species	Longest reproductive organ			Longest reproductive organ [%]		
		style	stamens	equal	style	stamens	equal
violet	48	40	6	2	83.3%	12.5%	4.2%
violet/white	22	14	8	0	63.6%	36.4%	0.0%
white	19	16	3	0	84.2%	15.8%	0.0%
**non-yellow**	**89**	**70**	**17**	**2**	**78.7%**	**19.1%**	**2.2%**
**yellow**	**17**	**7**	**9**	**1**	**41.2%**	**52.9%**	**5.9%**
all	106	77	26	3	75.5%	25.5%	2.9%

**Fig 5:**
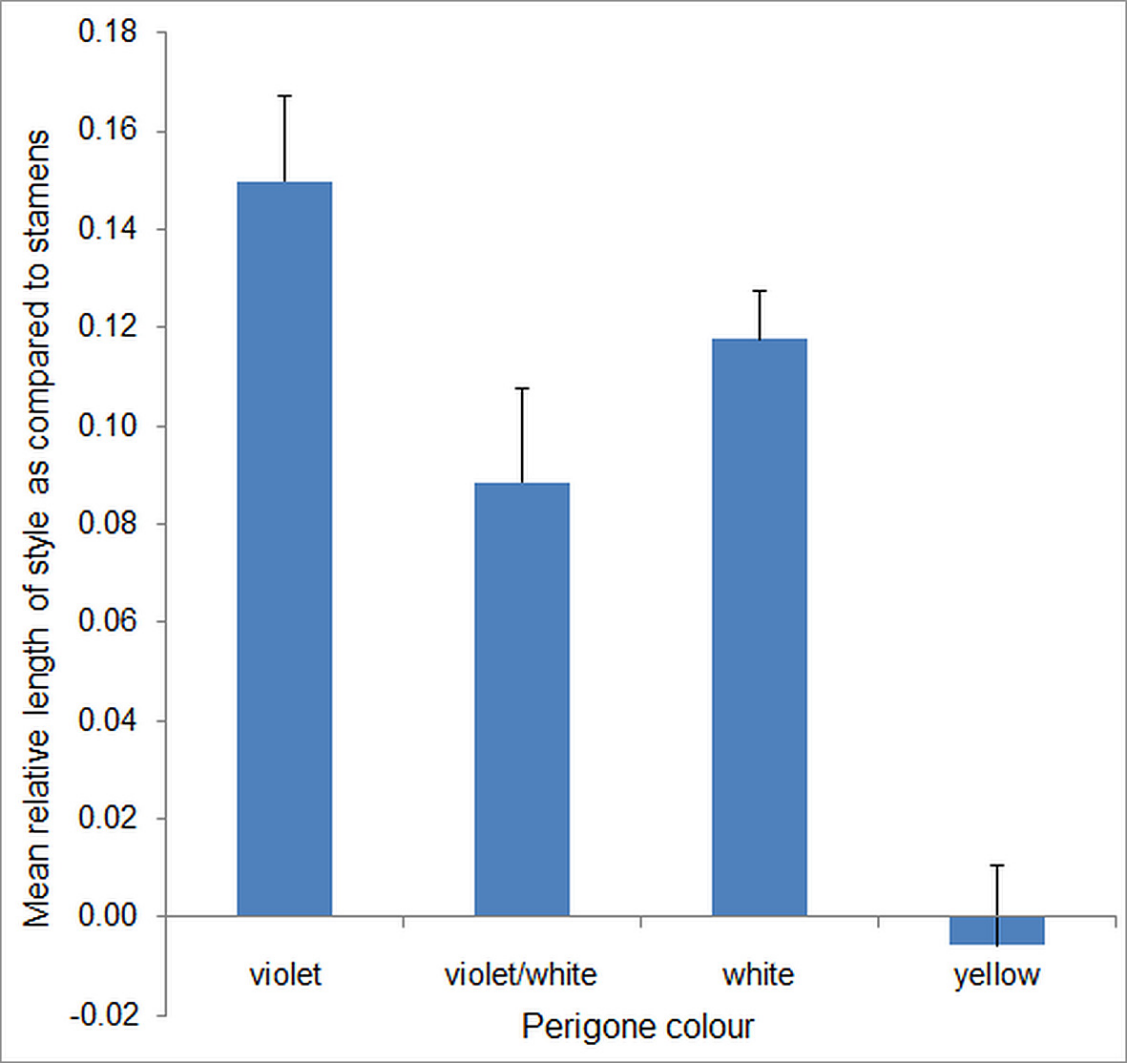
Mean relative length of style compared to stamens for *Crocus* species with different perigone colours. With standard error; n = 106 species of *Crocus* comprising 48 violet, 22 violet/white, 19 white and 17 yellow coloured species.

Information concerning the length ratio of style and stamens gathered from the monographs of Maw [38] and Mathew [22] for the most part corresponds to our measurements (S1 Table). Out of only 14 divergences, five coincide with our hypothesis, nine argue against it. Thus, the results seem to be relatively independent from the source of data. The linear regression analysis of the phylogenetic independent contrasts [39] indicates that the lengths of stamens and style are positively correlated (n = 106; adjusted R-squared = 0.048; p = 0.0134) and that the length of the style in relation to the length of the stamens is lower in yellow *Crocus* flowers than in white and in violet *Crocus* flowers (Fig 4). The linear regression analysis shows a significant positive correlation between relative style length and relative stamens length in violet (adjusted R-squared = 0.249, p = 0.0002) as well as white *Crocus* flowers (adjusted R-squared = 0.571, p = 0.0001), but not in violet or white (adjusted R-squared = 0.023, p = 0.235) and yellow *Crocus* flowers (adjusted R-squared = -0.060, p = 0.7579). The mean relative length of styles as compared to stamens for the 86 non-yellow *Crocus* species is significantly longer than the mean relative length of styles as compared to stamens for the 17 yellow Crocus species (t-Test, p>0.01, Fig 5).

**Fig 6:**
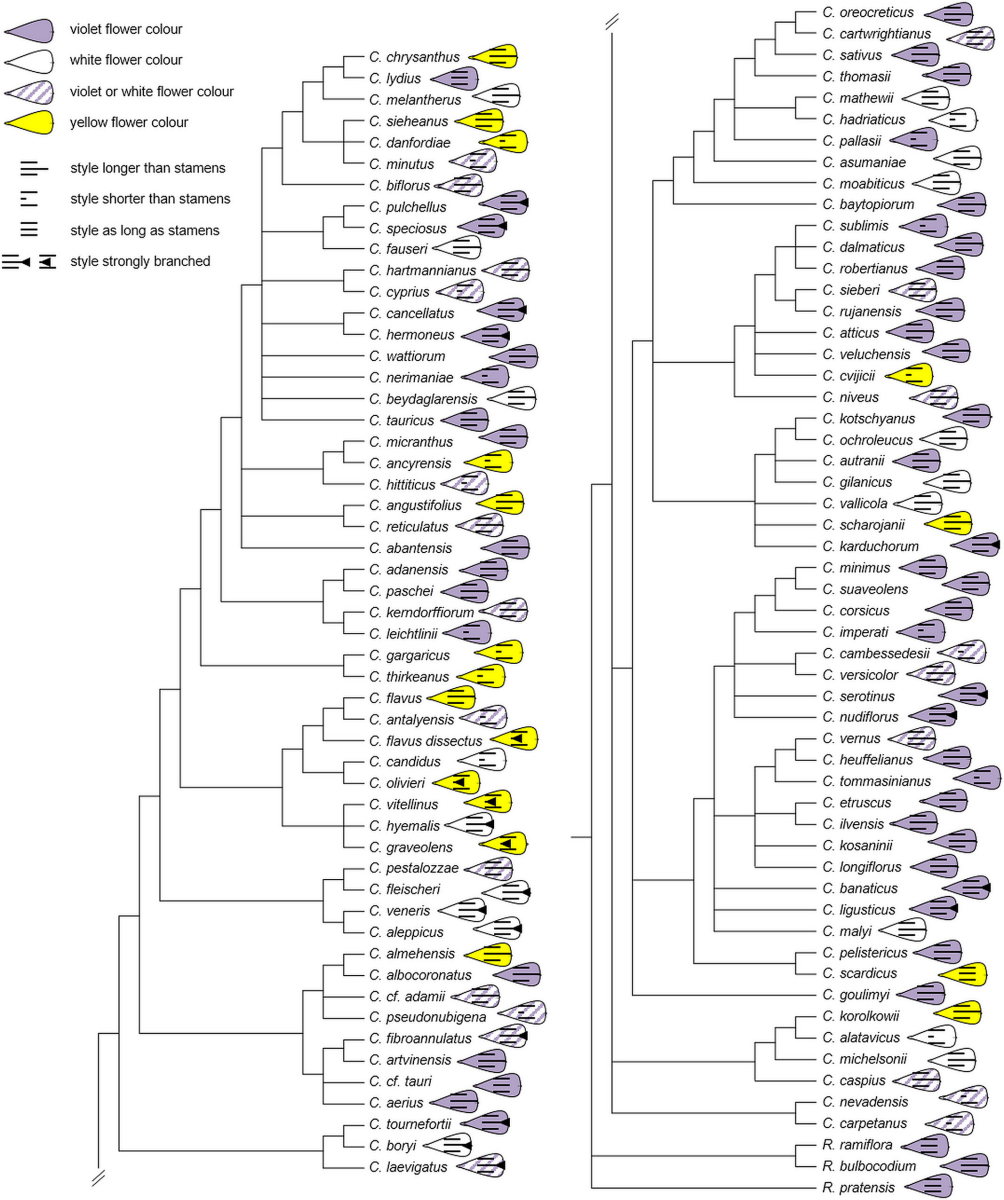
Phylogenetic tree of the genus *Crocus* with superimposed floral characters. Phylogenetic tree obtained by Bayesian phylogenetic inference of the nuclear rDNA ITS region. The floral characters indicated are flower colour and relative style length as compared to the relative stamens length and branching of the style for 106 *Crocus* and 3 *Romulea* species.

According to the phylogeny, the yellow tepal colour has evolved independently up to 12 times (Fig 6). Associated with evolutionary changes in perigone colour a reduction of style length occurs 4 times, whereas an elongation occurs once. In addition, branched styles have evolved independently up to 11 times and occur in 4 out of 17 yellow-flowering species and 17 out of 89 non-yellow-flowering species.

## Discussion

Stamen mimicry is a widespread and well described phenomenon [41,11,12]; the consequences of stamen mimicry on flower architecture and colour pattern have only rarely been studied [6,42,9]. Since flower-visiting bees target at stamens and stamen-mimicking signals of flowers [43] the flowers can facilitate the immediate pollen deposit on the stigma, if the stigma is mimicking visual and structural cues of stamens. Minimum requirements for bees to land on stigmas are that stigmas are salient and contrasting against the perigone as revealed by behavioural studies with artificial flowers [17,6]. This study addresses the suitability of the style in *Crocus* species for guiding pollinating insects towards the stigma for pollen deposition. Open *Crocus* flowers are bowl-shaped and offer a landing site in the form of their style and stamens protruding from the perigone. The colour contrast between style and perigone is essential for the function as a visual guide and the superior length of the style as compared to the stamens is essential for an achievement of pollen deposition before pollen uptake. Yellow and UV-absorbing *Crocus* flowers display little or no colour contrast of style and stamens against the perigone and thus are unlikely to guide landing insects towards an elongated style. In this study it was tested if blue and white *Crocus* flowers yellow possess elongated styles to achieve pollen deposition in the stigma in contrast to yellow and UV-absorbing *Crocus* flowers. There is some evidence from observations that the colour pattern provided by perigone and style of *Crocus* flowers is under selection [44]. The targeting of flower visitors when landing on a flower can facilitate cross-pollination. Flowers that are able to manipulate the flower visitors in a way that they touch the stigma before touching other floral organs probably benefit in terms of cross-pollination and reduction of self-pollination, particularly if the stigma is not touched again after the flower visitor has been dusted with pollen from that flower. *Crocus* flowers are textbook examples for upturned bell-shaped flowers with a more or less united column of style and stamens [45]. It is known that floral colour patterns play a dominant role in triggering the targeting of landing bees towards distinct sites [46–48,29]. Our results suggest that yellow-flowering *Crocus* species use a different pollination strategy as compared to violet- and white-flowering *Crocus* species. Elongated styles with three yellow stigmatic lobes offer a conspicuous and contrasting landing site in violet and white *Crocus* flowers, whereas the same structure is less conspicuous and less contrasting in yellow *Crocus* flowers for bee eyes if the reflectance in the ultraviolet range of wavelengths is similar. Our study, however, ignores hypothetical growth and elongation of tepals, styles and/or stamens during anthesis due to lack of data; it is unlikely that elongation of floral organs during anthesis will substantially change length relation between the organs.

The uniform ultraviolet absorbing properties of tepals, stamens and styles in the yellow-flowering *Crocus* species studied so far [25] and in this study suggest that also for bee eyes the perceptual contrast between these floral organs is low. The genus *Crocus* is prominent for flavonoids as floral pigments [49] except for the style, which also possesses carotenoids [23]. Yellow and UV-absorbing flavonoids are the pigments that colour the pollen wall of pollen in primarily wind-pollinated plants [41] and are thought to shield the chromosomes in the pollen grain against the mutagenic ultraviolet light [50]; the yellow and UV-absorbing flavonoids may thus represent the original floral colour signal present already before the onset of animal pollination [41,12]. The uniform absorption of ultraviolet light of the tepals of yellow-flowering members of the genus *Crocus* and species of the related genus *Romulea* (this study) and the genus *Sternbergia* (Avi Smida, personal communication) suggests evolutionary constraints in flower pigments.

The yellow perigone colour is neither a primitive character in *Crocus* nor an adaptation that has evolved only in a few branches according to the phylogeny (Fig 6). Moreover, most if not all yellow-flowering *Crocus* species have a non-yellow-flowering sister species. Transitions in flower colour are common among the angiosperms, with sister species frequently showing variation in colour hue [51]. These evolutionary colour transitions are often associated with pollinator transition [52]. Although pollinators of many *Crocus* species are not documented, the flowering syndrome within *Crocus* seems to be rather constant. The attractiveness for syrphid flies rather than bees might have played a role for the evolution of yellow flowers due to innate preferences for distinct colour hues [15].

Our results confirm the hypothesis that yellow styles might represent a less conspicuous landing signal in yellow-flowering *Crocus* species due to reduced colour contrast against the perigone. This hypothesis predicted our finding that yellow-flowering *Crocus* species might be less able to benefit from yellow stigmas as a landing guide for pollinators. Our results show that yellow-flowering *Crocus* species are less likely to possess elongated styles. How can these flowers with short styles assure guidance of pollinators towards the style? In *C*. *siehanus* the style is of a deep orange colour and thus might be more attractive than the yellow corolla and the yellow stamens. In *C*. *scharojanii* the flowers do not open fully and by this way might mechanically ensure the contact between the pollinator’s body and the stigma when the pollinator squeezes into the perigone. In *C*. *olivieri* and *C*. *flavus* subsp. *dissectus* the multi-branched stigma might increase the chance of stigma contact when the pollinator crawls into the flower. Moreover, the short-styled species might use a fundamentally deviant strategy and benefit from facilitated self-pollination established by pollen grains falling from long stamens onto the stigmas of a short style; however, self-compatibility has not been demonstrated in these species. Indeed, there is little knowledge about how the shape of *Crocus* flowers in conjunction with the opening and closing of the perigone [45] might support pollination. If the tepals fully open then the style and stamens are a prominent target; however, if the perigone opens only slightly then flower visitors have to land on the outside of the tepals and crawl into the flower. When foraging/nectar-seeking bees crawl along the tepals into the floral tube, only the dorsal side of the bee is dusted with pollen and subsequent landing on the style of another conspecific flower will not result in an effective pollen transfer.

Attraction of pollinators seemingly is not the sole function of floral pigments in members of the genus *Crocus*. The flower colour also plays a specific role in autumn-flowering *Crocus* flowers; violet and white flowers are able to trap heat energy better than yellow ones due to reflectance properties of the inner tepal surfaces [32]. There are probably strong constraints on the architecture and colouring of style, stigmatic lobes, stamens, anthers and pollen in flowers of *Crocus* species. Emergency self-pollination through falling pollen grains is excluded if the style is longer than the stamens. Protection of pollen deposited on the stigma against sun, wind, and cold air should be better with short and less exposed styles. The results of this study show that *Crocus* flowers do not display an ultraviolet bull’s eye resulting in minor colour contrast between style and perigone in yellow *Crocus* species and suggests that this is crucial for the function of the style as a contrasting, visually attractive landing platform for pollinators.

## Supporting Information

S1 Table List of 106 species of *Crocus* studied. The list indicates the perigone colour in 4 categories, the style length in relation to the perigone length, the stamen length in relation to the perigone length, the style length in relation to the stamen length, and floral characteristics like branched styles and colour of stamens and styles. For comparison, the classification in categories for style and stamen (or equal) length is listed as stated by Maw [38] and Mathew [22] respectively. Information regarding markings on the inside or outside of the tepals may be incomplete due to limitations in availability of reliable photographs. The image sources of photographs used are listed.
(XLSX)
